# APP21 transgenic rats develop age-dependent cognitive impairment and microglia accumulation within white matter tracts

**DOI:** 10.1186/s12974-018-1273-7

**Published:** 2018-08-28

**Authors:** Nina Weishaupt, Qingfan Liu, Sheojung Shin, Ramandeep Singh, Yuksel Agca, Cansu Agca, Vladimir Hachinski, Shawn Narain Whitehead

**Affiliations:** 1 Vulnerable Brain Laboratory, Department of Anatomy and Cell Biology, Schulich School of Medicine and Dentistry, University of Western Ontario, 1151 Richmond St, London, Ontario N6A 5C1 Canada; 2 Department of Veterinary Pathobiology, College of Veterinary Medicine, University of Missouri, Columbia, MO USA; 3 Clinical Neurological Sciences, London Health Sciences Centre, University of Western Ontario, London, Ontario N6A 5A5 Canada

**Keywords:** Microglia, White matter inflammation, APP21 transgenic rat, Cognitive impairment, Alzheimer’s disease, Rat model

## Abstract

**Background:**

Most of the animal models commonly used for preclinical research into Alzheimer’s disease (AD) largely fail to address the pathophysiology, including the impact of known risk factors, of the widely diagnosed sporadic form of the disease. Here, we use a transgenic rat (APP21) that does not develop AD-like pathology spontaneously with age, but does develop pathology following vascular stress. To further the potential of this novel rat model as a much-needed pre-clinical animal model of sporadic AD, we characterize APP21 transgenic rats behaviorally and histologically up to 19 months of age.

**Methods:**

The open field test was used as a measure of activity; and the Morris water maze was used to assess learning, memory, and strategy shift. Neuronal loss and microglia activation were also assessed throughout the brain.

**Results:**

APP21 transgenic rats showed deficits in working memory from an early age, yet memory recall performance after 24 and 72 h was equal to that of wildtype rats and did not deteriorate with age. A deficit in strategy shift was observed at 19 months of age in APP21 transgenic rats compared to Fischer wildtype rats. Histologically, APP21 transgenic rats demonstrated accelerated white matter inflammation compared to wildtype rats, but interestingly no differences in neuron loss were observed.

**Conclusions:**

The combined presence of white matter pathology and executive function deficits mirrored what is often found in patients with mild cognitive impairment or early dementia, and suggests that this rat model will be useful for translationally meaningful studies into the development and prevention of sporadic AD. The presence of widespread white matter inflammation as the only observed pathological correlate for cognitive deficits raises new questions as to the role of neuroinflammation in cognitive decline.

**Electronic supplementary material:**

The online version of this article (10.1186/s12974-018-1273-7) contains supplementary material, which is available to authorized users.

## Background

To study the impact of the prodromal phase of Alzheimer’s disease (AD), including the role of potential risk factors in the progression of AD, novel preclinical models are needed. As rodents do not develop signs of AD naturally, different models have been created to exhibit AD-like pathology, including the introduction of amyloid fragments [1], toxins [2–4], and one or more transgenes [5, 6]. Transgenic mice have been widely used to model pathological correlates of AD, however, most do not fully recapitulate the pathological and behavioral components of AD and therefore have demonstrated limited translational success [5–7]. A small number of transgenic rat models for the study of AD have only more recently become available [8, 9]. Rats may be more valuable models than mice as they are both physiologically closer to humans [7], and are better than mice at performing complicated cognitive tasks, such as those requiring complex executive functions like strategy shift [10], a behavioral precursor to memory impairment more commonly associated with AD.

Here, we use behavior and histology, to characterize the transgenic (APP21) rat model for its pre-clinical use in studying mild cognitive impairment (MCI) and the early-phase of sporadic AD. These transgenic rats were created on a Fisher 344 background and express the human amyloid precursor protein (APP) gene with both Swedish and Indiana mutations [11]. As reported previously, these rats do not develop histological hallmarks of AD spontaneously when aged up to 30 months [12]. They do, however, develop histopathological signs of AD when challenged with brain extract from AD patients injected intracerebrally [12], or when experimental hydrocephalus is induced [13]. To date, behavioral and histological characterizations of the APP21 transgenic rat are lacking. Therefore, in the present study, we compare the behavior and histopathology of the APP21 transgenic rat to wildtype Fischer rats over the course of 19 months to establish a solid basis for future experimentation using this model. We hypothesized that APP21 transgenic rats would demonstrate enhanced age-related learning and memory deficits due to the pathogenic human mutation expression. We observed deficits in executive function and a developing neuroinflammatory phenotype, with a focus on white matter tracts, suggesting that APP21 transgenic rats may closely model the clinical phase of mild cognitive impairment (MCI) [14, 15]. Therefore, APP21 rats may have unique potential for translationally meaningful research into risk factors and preventative approaches to non-familial forms of dementia [16].

## Methods

### Animals and experimental groups

**Fig. 1:**
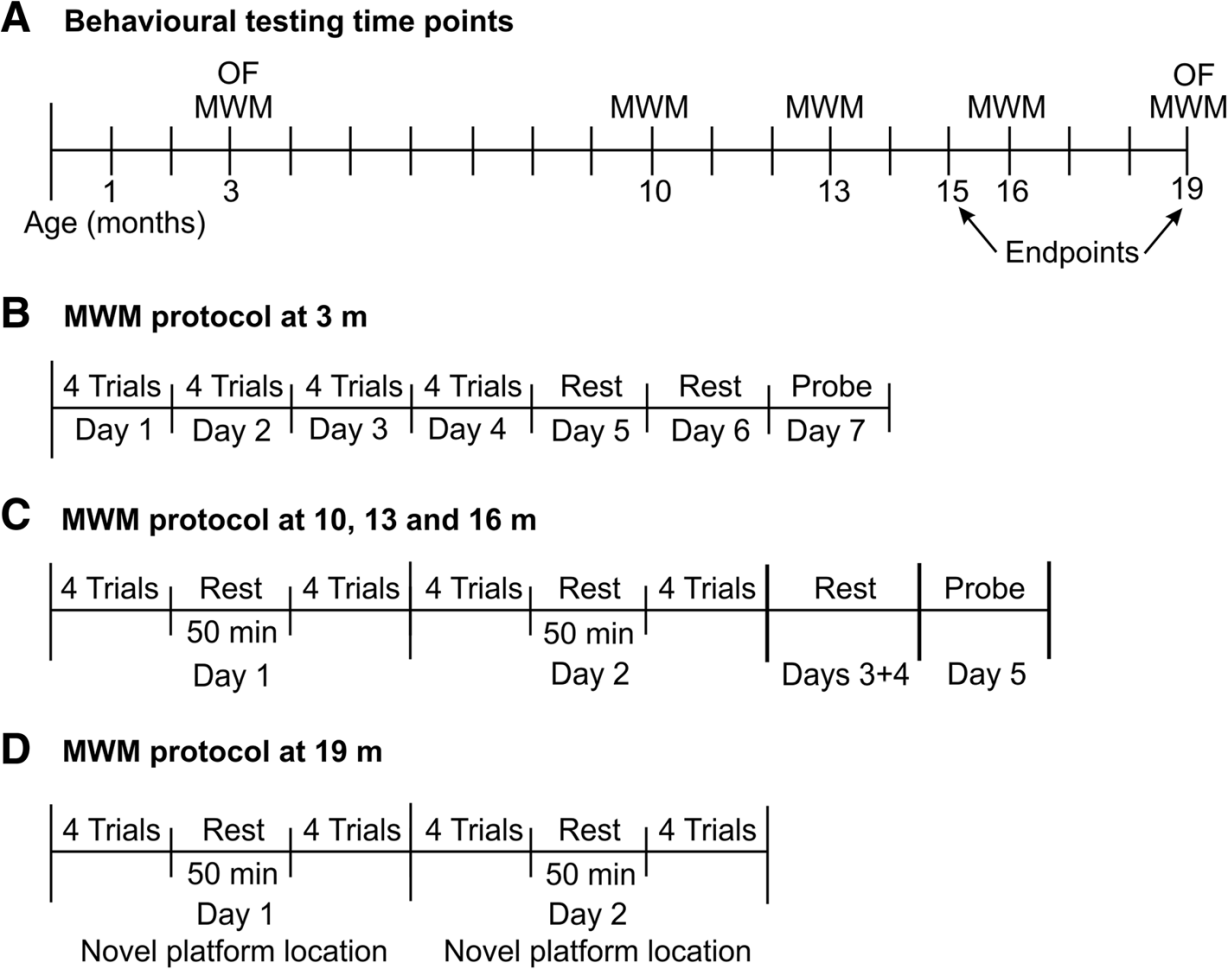
Overview of behavioral testing. Rats were assessed in the open field (OF) and in the Morris water maze (MWM) at different ages (**a**). The initial water maze protocol (**b**) consisted of 4 days of acquisition trials. Subsequent testing was done by more intensive training on two acquisition days (**c**). At 19 months of age, rats underwent a unique water maze protocol aimed at testing their mental flexibility in a strategy shift paradigm over two consecutive days (**d**)

All animal studies have been approved by local University Animal Use Committee (UWO 2014-016) and are compliant with the Canadian Council of Animal Care. Six cohorts of male Fisher 344 rats (total of 40) were bred in-house and aged to either 15 or 19 months (m), each cohort consisting of age-matched Fisher 344 wildtype (WT) and APP21 transgenic rats (TG). APP21 rats express the human APP gene with both the Swedish and Indiana mutations known to cause familial AD in humans [11]. Homozygous founder breeding pairs were kindly provided by Dr. Yuksel Agca (University of Missouri, [11]). Presence of the transgene in all offspring from homozygous pairings was confirmed by PCR. WT and TG rats underwent a behavioral testing schedule as shown in Fig. 1. Four additional rats (two WT and two TG) were euthanized at 3 months for histological processing of their brains. All rats were male, group housed, maintained at a 12 h/12 h light/dark cycle, and fed ad libitum.

### Behavioral assessments

#### Open field

Rats were allowed to freely move in an Open Field Test Arena (MedAssociates Inc., St. Albans, USA, 45 cm × 45 cm) for 10 min at 3 months of age. Movement in the arena was automatically tracked by three 16-beam infrared arrays and recorded by Activity Monitor Software (MedAssociates Inc.). The ambulatory time and the number of rears were used to assess the rats’ activity level. The test was repeated at 19 months of age using ANY-maze software-based tracking from a video camera.

#### Morris water maze

Spatial learning, memory, and strategy shift ability, three distinct cognitive domains often impaired in individuals with MCI or early dementia, were evaluated. Rats were introduced into a circular pool, 146 cm in diameter, filled with water rendered opaque by water-soluble, nontoxic, black paint.

Spatial learning: at 3 months of age, rats were trained (four trials per day for four consecutive days) to swim to a submerged, invisible platform (Fig. 1b). For each learning trial, rats were introduced in the pool from a different quadrant and were given 60 s to find the platform (location remained the same). Once on the platform, rats were left in place for 30 s to allow for spatial orientation based on light/dark cues on the walls around the pool. Rats that did not reach the platform within the time limit were guided to the platform by the experimenter. At the 10-month, 13-month, and 16-month time points, 8 re-acquisition trials were conducted on 2 consecutive days for learning of a new platform location (platform location remained the same across the 16 trials, Fig. 1b). Re-acquisition trials were run following the same protocol as learning trials in blocks of four trials. A second block of four trials was started 50 min after each rat’s fourth learning trial on the same day (Fig. 1c). Thus, working memory could be assessed within blocks of four trials (30 s platform orientation time equals the delay between four consecutive trials), short-term memory could be assessed between blocks of four trials (50 min delay between trial 4 and trial 5, and between trial 12 and 13) and 24-h memory could be assessed between the first and second day of re-acquisition (trial 9).

Long-term memory recall: memory retention was tested by a 30 s spatial probe trial, 3 days following the last learning trial (Fig. 1b, c), where the platform was removed from the pool. All rats were released from the same location.

Strategy shift: at 19 months of age, the rats’ ability to shift strategies from 1 day to the next was tested using a modified re-acquisition paradigm. On day 1, rats received two blocks of four learning trials to learn a new platform location and establish a memory of the location. On day 2, the platform location changed again and rats were given two blocks of four learning trials to learn the new platform location (Fig. 1d). This way, rats were asked to establish an accurate memory of a specific platform location on day 1, and then to recognize that the old location was no longer valid, and respond with a shift to a search strategy for a new location on day 2. At the end of this assessment, all rats underwent four cued trials, in which the platform was rendered visible by a yellow marker that reached above the water surface. The platform location was changed for each of these cued trials.

Morris water maze analysis: for all trials, the rats’ movements within the pool were tracked using a webcam and analyzed by ANY-maze software (Stoelting Co., Wood Dale, USA). Learning and re-acquisition were assessed by plotting the latency to reach the platform, while probe trial performance was measured by calculating the percentage of time the rat spent in the quadrant in which the platform was previously located (target quadrant). In addition to these automated out-reads, we manually performed a swim pattern analysis and expressed the percentage occurrence of “direct swim” (DIM), “chaining” (CSS), and search strategy, for each rat [17, 18].

### Perfusion and tissue preparation

Rats were euthanized with an overdose of pentobarbital (Euthanyl), transcardially perfused with 10 mM phosphate-buffered saline (PBS) containing 5000 IE/L heparin (Pharmaceutical Partners of Canada, Richmond Hill, ON, Canada) followed by 4% paraformaldehyde. Brains were harvested, post-fixed in 4% paraformaldehyde for 24 h, and subsequently transferred into a 30% sucrose solution. Each brain was then sectioned into 10 series of 30-μm-thick sections, collected free-floating in cryoprotectant (30% sucrose, 30% ethylene glycol in 10 mM PBS), and stored at − 20 °C.

### Histology immunohistochemistry

Free-floating sections were first incubated in 1% H_2_O_2_ in 0.01 M PBS for 15 min. Sections were then blocked at room temperature in a solution containing 10% horse serum and 0.01 M PBS for 1 h. Sections were then incubated overnight at 4 °C in either mouse anti-NeuN (1:1000, EMD Millipore, Etobicoke, ON, Canada), rabbit anti-IBA-1 (1:1000, Wako Chemicals USA Inc., Richmond, VA, USA), or mouse anti-OX-6 (1:1000, BD Pharmingen, Mississauga, Canada) primary antibodies. Following 3 × 10 min washes in 0.01 M PBS, sections were incubated in biotinylated anti-mouse secondary antibody (1:2000, Vector Laboratories Inc., Burlingame, CA, USA) for 1 h at room temperature and then incubated in 2% avidin-biotin complex solution (Vectastain Elite ABC Kit, Vector Laboratories, Inc., Burlingame, CA, USA) for 1 h at room temperature. Finally, sections were incubated in 0.05% 3,3′-diaminobenzidine tetrahydrochloride (DAB; Sigma) and 1% H_2_O_2_ for approximately 1–3 min at room temperature. Sections were dehydrated in ascending concentrations of alcohol, submerged in xylene, and then mounted with Depex mounting medium (Depex, BDH Chemicals, Poole, UK). Mounted brain sections were also processed with Luxol fast blue to detect changes in myelin content [19].

### Histological analyses

Investigators were blinded to all histological analysis and measurements. Stained sections were analyzed using a Nikon Eclipse Ni-E upright microscope with a Nikon DS Fi2 color camera head (NIS Elements Imaging, Nikon Instruments, Melville, NY, USA). All integrated density measurements were calculated using ImageJ software (NIH Software) and were averaged from three adjacent tissue sections per region per rat. Integrated density measurements were made within white matter tracts and the cerebral cortex of IBA-1 (general microglia marker) immunostained brain sections. Integrated density measurements were made within white matter tracts for OX-6 (marker for activated microglia) immunostained sections. OX-6 immunoreactivity was assessed in the cerebral cortex of each rat and was rated on a 0–3 point scale. Neuron loss was assessed using NeuN cell counts from both dorsal and lateral cortices (left and right) from three adjacent sections per rat. Total NeuN counts within the regions of interest were calculated using particle analysis within Nikon Elements software. Integrated density measurements were made from gray-scale converted Luxol fast blue photomicrographs from three adjacent tissue sections per region per rat.

### Statistical analysis

The number of analyzed rats (*n*) included in individual analyses varies throughout this study primarily due to mortality for a variety of health issues not related to the experimentation (17.5%). All comparisons among groups at different time points were analyzed using two-way ANOVA followed by Sidak’s multiple comparisons where indicated. Open field activity was compared using a Mann–Whitney test. Histology was analyzed using two-way ANOVA followed by Tukey’s post-hoc tests. Data are expressed as group mean ± SEM and *n* values are indicated on individual graphs or within graph bars.

## Results

### TG rats take longer for spatial learning due to a working memory deficit

**Fig. 2:**
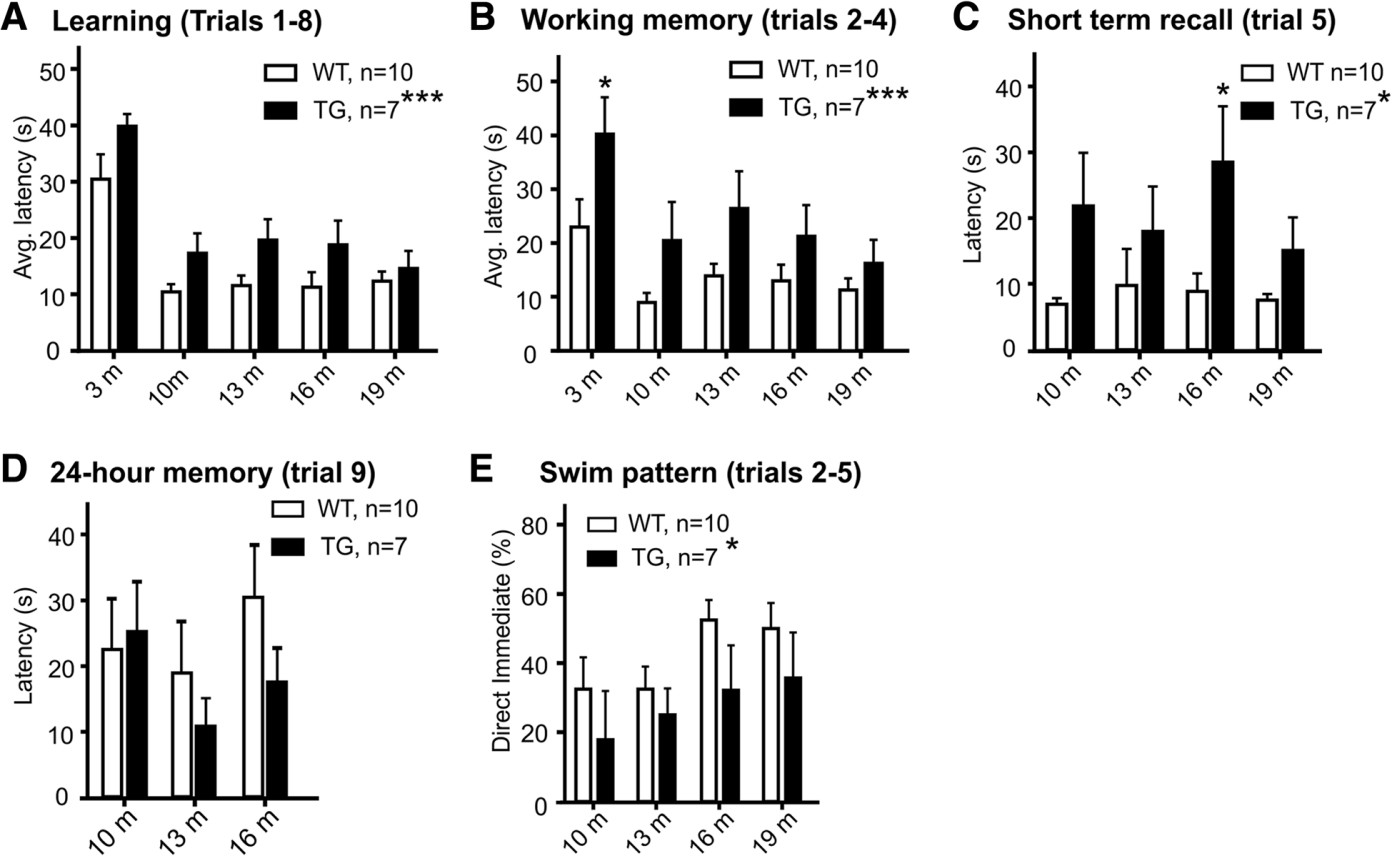
Performance in Morris water maze acquisition across age. To assess how fast TG and WT rats learned a new platform location in the MWM task, the average latency to find the platform in the first eight learning trials was calculated at each age time point (**a**). TG rats were significantly slower in finding the platform than WT rats across time points. To assess working memory performance, the latency to find the platform in trials 2–4, which were 30 s apart, was compared between genotypes (**b**). TG rats demonstrated worse performance in working memory than WT rats. Comparing performance in trial 5 was used as a measure of memory as all rats had completed four learning trials 50 min prior (**c**). A significant effect of genotype was observed in trial 5, and TG rats performed significantly worse at 16 months of age. 24-h memory performance in trial 9 was not significantly different between genotypes (**d**). Swim pattern analysis showed that TG rats exhibited the “direct immediate” swim pattern (DIM), a sign of accurate memory, significantly less often than WT rats in trials 2–5 (**e**). Graphs show mean ± SEM. One asterisk indicates *p* < 0.05, three asterisks indicate *p* < 0.001 (two-way ANOVA results indicated beside genotype and results of Sidak’s multiple comparisons indicated on bars)

All rats were given 16 acquisitions to learn the location of a submerged platform in the Morris water maze task. WT and TG rats were able to learn the location to a similar extent by the end of the acquisition period; however, TG rats did not learn the platform location as quickly as WT rats. This was seen in a consistent difference in trial latencies between the genotypes in the first eight trials of each acquisition session (*p* = 0.0006, two-way ANOVA, Fig. 2a), while swim speeds were comparable between the two groups across learning trials (Additional file 1: Figure S1). To assess the rats’ working memory performance, the average latency of trials 2–4 was plotted at each point in time when the rats were introduced to a new platform location. Trial 1 was excluded as no memory is involved in finding the new platform location in the first trial. Trial 5 was excluded for this analysis as it was conducted 50 min or 24 h (at 3 m of age) after the first set of four trials. A two-way ANOVA across all time points revealed a significant effect of genotype (*p* = 0.0003, Fig. 2b), with TG rats consistently taking longer to find the platform than WT rats. Sidak’s multiple comparisons revealed a significant difference only at the 3-month time point (*p* < 0.05). As a two-way ANOVA does not allow the data from rats that did not reach the 19 months endpoint to be included, we also looked at individual time points using a Mann–Whitney test and increased statistical power. Using this method, a significant difference between the two groups was still evident at 10 months of age (20.02 ± 15.13, *n* = 12, vs. 10.65 ± 7.90, *n* = 11, *p* = 0.046).

Short-term memory was assessed by comparing trial latencies for trial 5. Trial 5 requires the rats to remember what they learned 50 min before in four consecutive acquisition trials. As trial 5 was conducted 24 h after trial 4 at 3 months of age, this assessment was only done from 10 months of age onwards. Similar to the working memory result, TG rats took consistently longer to find the platform in trial 5 than WT rats across all time points (21.86 vs. 6.89 at 10 months, 17.99 vs. 3.75 at 13 months, 28.53 vs. 8.86 at 16 months, 15.07 vs. 7.02 at 19 months, *p* = 0.0058, Two-way ANOVA, Fig. 2c). A Sidak’s multiple comparison test revealed a significant difference between the groups at the 16 m time point (*p* < 0.05). The 24 h memory between trials 8 and 9 was likewise assessed, with no significant differences between the genotypes in latencies in trial 9 (Fig. 2d).

In conclusion, TG rats took longer to build spatial memory due to working and short-term memory deficits when compared to their WT counterparts. This was confirmed by a swim pattern analysis, which showed that WT rats have significantly more “direct swim” patterned trials than TG rats in trials 2–5 (*p* = 0.0392, two-way ANOVA, Fig. 2e); an indicator for accurate memory.

### TG rats have no long-term memory recall deficit

**Fig. 3:**
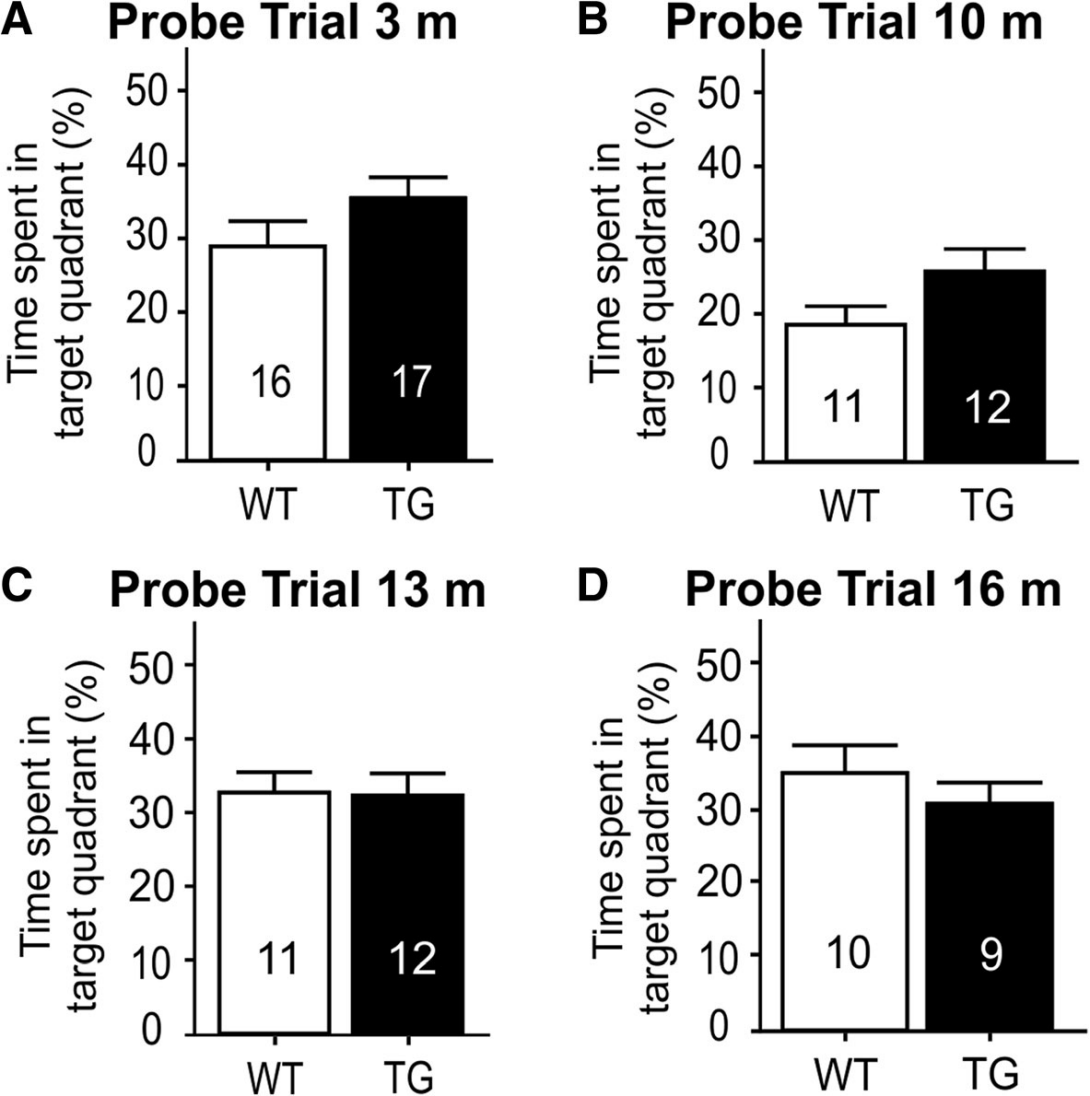
Memory performance in probe trials across age memory recall performance was assessed in a probe trial 3 days after the completion of 16 acquisition trials, and no statistically significant difference was found between the genotypes at any age. The number of rats analyzed is stated in each bar. **a** Probe trial, 3 months; **b** probe trial, 10 months; **c** probe trial, 13 months; **d** probe trial, 16 months

The rats’ 3-day memory was assessed in a probe trial, where the time spent in the target quadrant was assessed. Both genotypes performed equally (Fig. 3), and their performance did not decrease up to 16 months of age, the last time point that long-term memory was assessed. Most values show, however, that both rat groups performed only 5 to 10 percentage points above chance (25%), indicating that memory recall was not as robust as often seen in young healthy rats [20].

### TG rats demonstrate a deficit in the ability to shift strategy

**Fig. 4:**
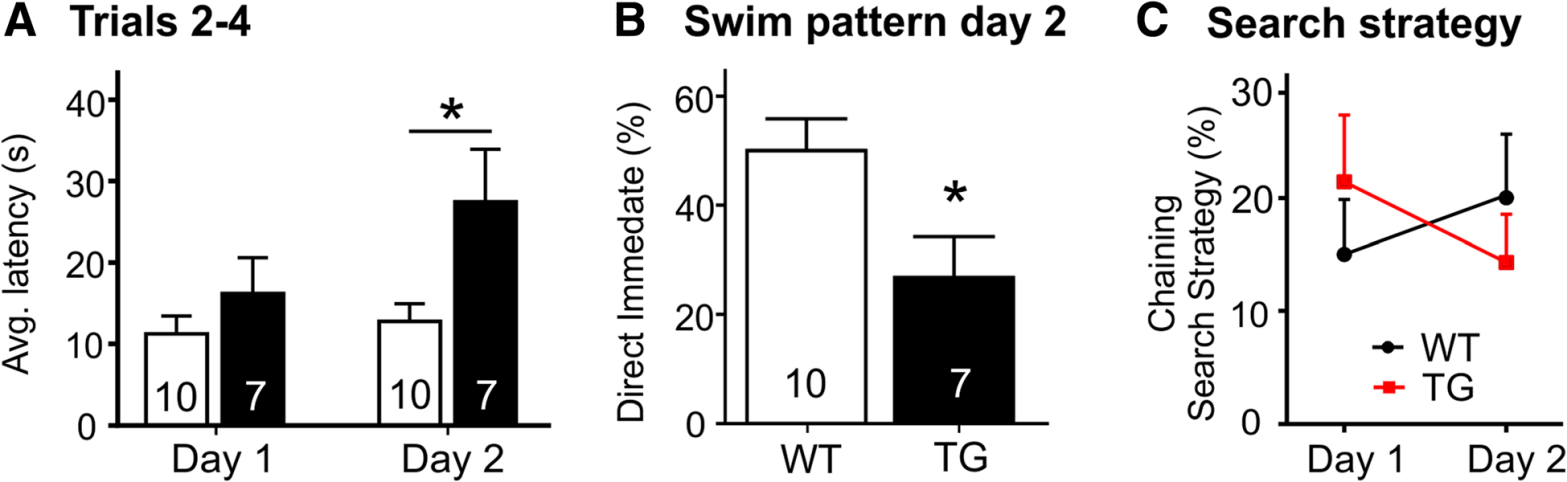
Strategy shift performance in the Morris water maze. A significant effect of genotype was observed when assessing latency to find the hidden platform (**a**). A lack of accurate learning and memory in TG rats was also reflected in TG rats exhibiting significantly less “direct swim” patterns (DIM) during the learning of the novel platform location on day 2 (**b**). WT animals increased the use of a search strategy from day 1 to day 2, while TG rats exhibited a decrease in the chaining search strategy (**c**)

At 19 months of age, all rats performed a strategy shift water maze task. As before, they were asked to learn a new platform location in two blocks of eight trials on day 1, but this time the platform location was unexpectedly changed on day 2. While TG rats (16.22 s ± 11.63) were on average 5 s slower than WT counterparts (11.25 s ± 6.96) at finding the platform during trials 2–4 on day 1, they were running on average almost 15 s behind WT rats on day 2 with the changed platform location (27.46 ± 17.11 vs. 12.77 ± 6.84, *p* = 0.0131, two-way ANOVA effect of genotype, Fig. 4a). TG rats were thereby significantly slower in shifting focus to the new platform location than WT rats (*p* < 0.05, Sidak’s multiple comparisons), who only showed minor delays in finding the platform on day 2 compared to day 1 (Fig. 4a). The significant difference between the groups in successfully adjusting to the novel platform location on day 2 was also reflected in the percentage of direct swim (DIM) pattern occurrences, which was significantly higher in WT (50.00 ± 18.63) than in TG rats (26.79 ± 19.67, *p* = 0.0286, Mann–Whitney, Fig. 4b). Interestingly, a change in swim pattern was observed in both groups of rats between days 1 and 2 of the strategy shift paradigm. While TG rats were using the chaining search strategy (CSS) more than WT rats on day 1, this relation reversed on day 2 (Fig. 4c). With TG rats using alternative swim patterns, WT rats seemed to show an appropriate adjustment by employing a search strategy more often than TG rats.

### TG rats show less exploratory behavior in the open field arena

WT rats spent significantly more time actively exploring an open field arena than TG rats at 3 months, a difference that persisted at 19 months of age (Mann–Whitney test, Additional file 1: Figure S2).

### TG rats develop widespread white matter inflammation sooner than WT rats

**Fig. 5:**
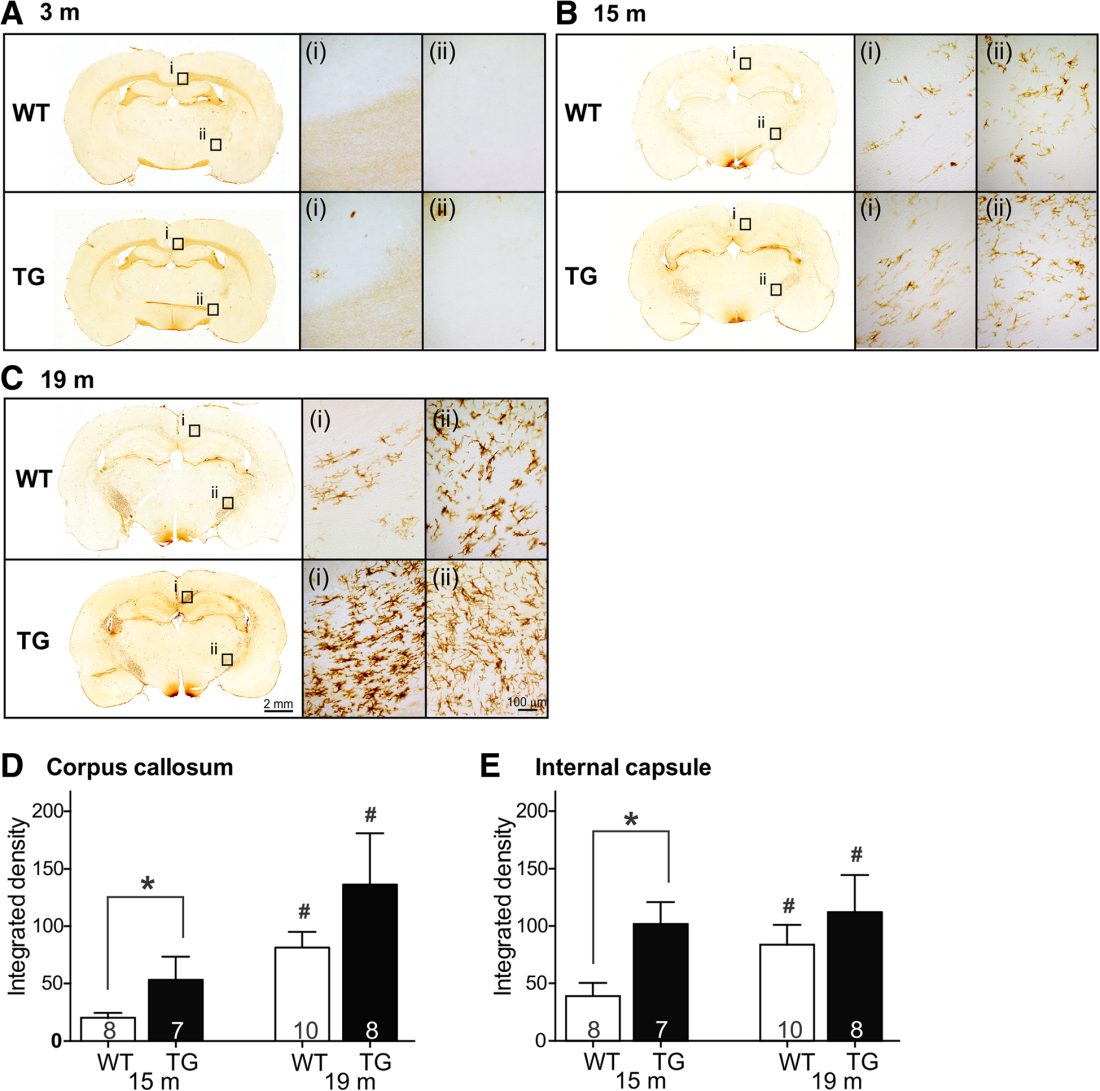
Activated microglia within white matter tracts: At 3 months of age, OX-6 positive activated microglia were sparse/rare within the corpus callosum and internal capsule (**a**). At 15 months of age, significantly more OX-6 positive signal was found within the corpus callosum of TG than WT rats (**b**). At 19 months of age, there were no statistical significant differences between activated microglia within the corpus callosum or internal capsule between WT and TG rats (**c**). The amount of OX-6 positive microglia in both white matter tracts increased visibly at 15 months, especially in TG rats (**b**). Activation of microglia in white matter continues to increase to 19 months of age, with comparable amounts in both genotypes (**c**). Quantification of OX-6 positive activated microglia within the white matter tracts showed a significantly higher load in TG vs. WT rats at 15 months, but not at 19 months in both the corpus callosum and (**d**) and internal capsule (**e**). Data is represented as group mean ± SEM. An asterisk indicates statistical significance between WT and TG rats, the number sign indicates statistical significance between 15 and 19 months WT or TG rats, respectively (*p* < 0.05, two-way ANOVA, Tukey’s post hoc test, *n* values indicated within bars on graphs)

OX-6-positive (MHC-II-expressing) cells are considered activated microglia and are a vital component of neuroinflammation. The clear majority of OX-6 positive cells were consistently observed within major white matter tracts, such as the corpus callosum, the internal capsule, and the anterior commissure (Fig. 5b-e). We confirmed that this histopathology developed over time by screening the brains of 3-month rats, which showed only sporadic OX-6 positive cells in any white matter tract (Fig. 5a). Due to the sporadic pattern of staining OX-6 positive activated microglia could not be accurately quantified in 3-month rats. We did however quantify OX-6 positive activated microglia in the corpus callosum and internal capsule of 15-month WT and TG rats. At 15 months of age, TG rats displayed a significant increase in activated microglia in both the corpus callosum and internal capsule compared to WT rats (*p* < 0.05, two-way ANOVA, Tukey’s post hoc test, Fig. 5b, d, e). By 19 months, the difference between genotypes was no longer observed (Fig. 5c–e); however, both WT and TG 19-month old rats demonstrated significantly higher amounts of activated microglia in both the corpus callosum and internal capsule compared to 15-month old WT and TG rats (*p* < 0.05, two-way ANOVA, Tukey’s post hoc test, Fig. 5d, e).

**Fig. 6:**
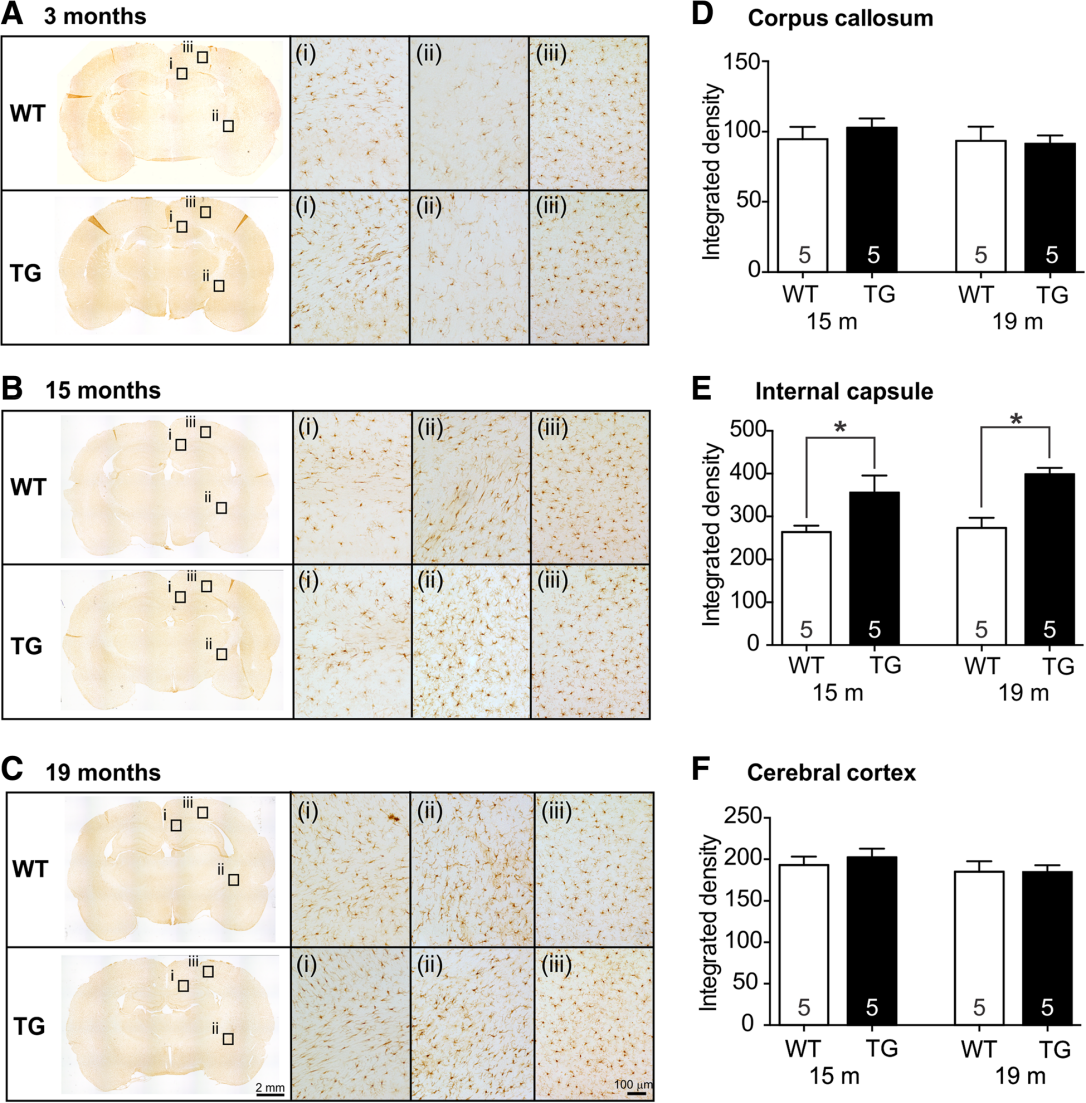
Total microglia analysis. IBA-1 positive microglia were assessed in 3 months (**a**), 15 months (**b**), and 19 months (**c**) WT and TG rats. Higher magnification insets are from the (i) corpus callosum, (ii) internal capsule, and (iii) cerebral cortex. Quantification shows TG rats have increased IBA-1 positive microglia in the internal capsule at 15 and 19 months of age (**d**), but no increased in IBA-1 positive microglia within the corpus callosum (**e**) or cerebral cortex (**f**). An asterisk indicates statistical significance between WT and TG rats, (*p* < 0.05, two-way ANOVA, Tukey’s post hoc test, *n* values indicated within bars on graphs)

To assess whether TG rats displayed increased levels of total microglia, IBA-1 immunostaining was performed, and white matter tracts and cortical regions were analyzed (Fig. 6). A significant increase in IBA-1 positive microglia was observed in both 15 and 19-month TG rats within the internal capsule (*p* < 0.05, two-way ANOVA, Tukey’s post hoc test, Fig. 6d, e), while no genotype differences were observed within the corpus callosum or cerebral cortex (Fig. 6f).

### Aged TG and WT rats show mild cortical inflammation but no differences in cortical neuron loss or myelin content

**Fig. 7:**
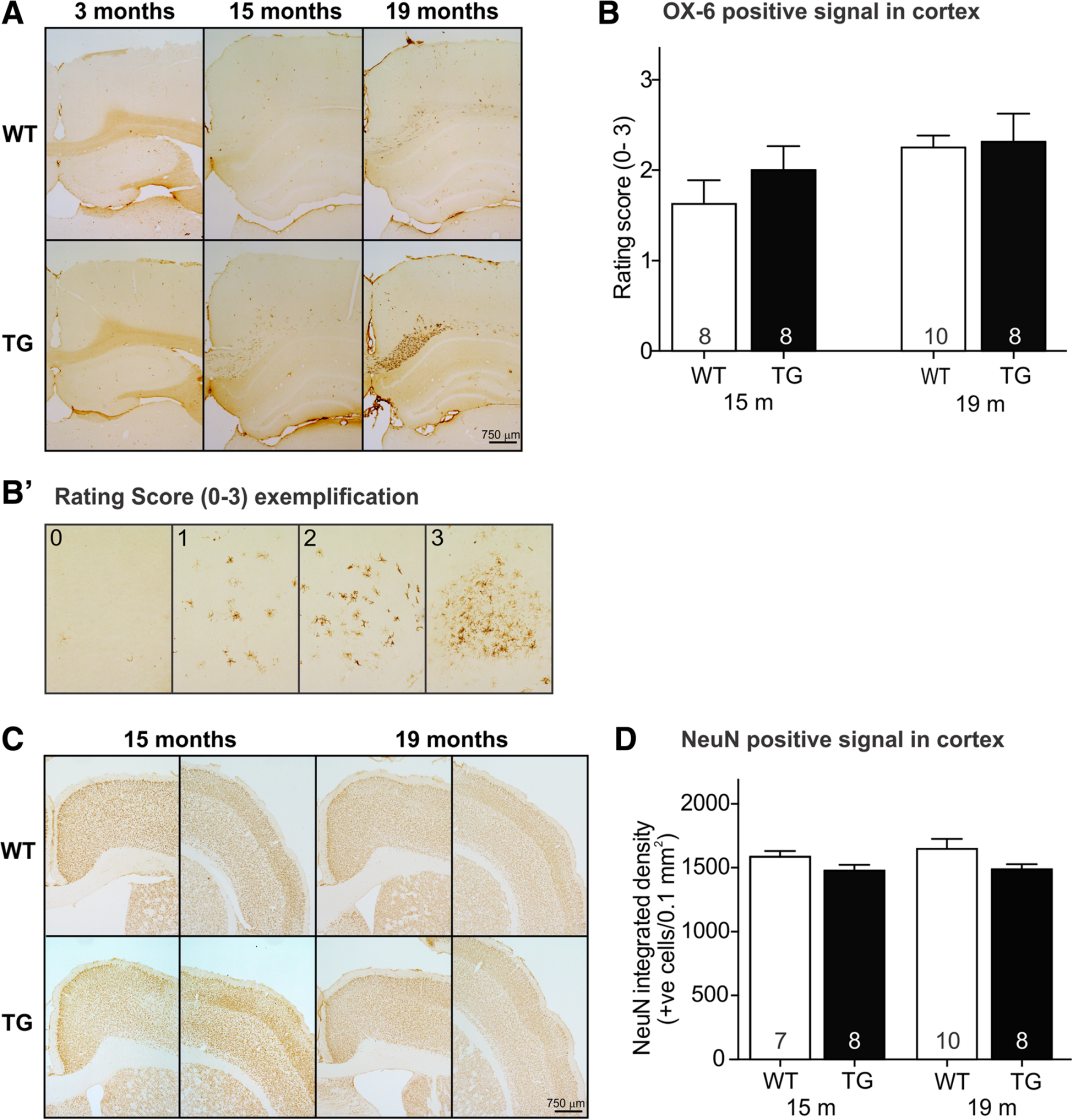
Cortical histopathology. Photomicrographs of cortical tissue sections from rats at different ages stained with OX-6 for activated microglia (A). Rating scores of OX-6 positive signal revealed a slight increase in cortical signal from 15 to 19 months of age, but no statistically significant genotype differences (B, rating score exemplified in B′). Photomicrographs of medial (left) and lateral (right) cortex sections stained for NeuN positive neurons (C). No genotype difference or neuronal loss was observed after quantification of NeuN signal (D). Statistical analysis was performed using a two-way ANOVA

When screening brains for OX-6 signal patterns, mild accumulations of OX-6 positive cells in the cortex were observed (Fig. 7A, B; rating scale exemplified in B′). Cortical activated microglia were present in aged WT and TG rats to a similar extent. In order to find out whether the white matter pathology and sporadic focal accumulation of cortical activated microglia resulted in neuronal loss, we quantified NeuN positive cells within the cortex of WT and TG rats (Fig. 7C, D). At 15 months of age, there were no statistically significant differences in NeuN-positive cells in the cerebral cortex. Likewise, there were no statistical differences between NeuN-positive cells in the cerebral cortex of 19-month rats. To determine if the increase in activated microglia present within the white matter tracts resulted in demyelination, Luxol fast blue was performed in 3, 15, and 19-month-old rats (Additional file 1: Figure S3). Quantification demonstrated no genotype- or age-related differences in myelin content (Additional file 1: Figure S3).

## Discussion

Sporadic AD has been difficult to model in laboratory rodents, as they do not naturally develop hallmarks of AD, such as amyloid plaques and neurofibrillary tangles. This is emphasized by the fact that the expression of two APP mutations by APP21 transgenic rats, each of which will result in familial AD in humans, does not result in amyloid plaque formation in old APP21 transgenic rats [12]. The expression of the APP transgene in these rats therefore does not induce what can be considered “rat AD,” instead, it merely renders these rats capable of developing AD-like pathology when challenged [12, 13]. As demonstrated in the present study, these rats do, however, present with spontaneous cognitive deficits, especially executive function deficits such as working memory and strategy shift ability. This finding supports accumulating evidence that amyloid plaques themselves may not be a good indicator of disease severity, and that soluble forms of amyloid may indeed be more neurotoxic and clinically meaningful [21]. It is interesting that the transgene by itself does not seem to produce a progressive deficit in spatial working memory or long-term memory, as measured here in the Morris water maze task over the course of 19 months. Strategy shift ability was only tested once at the end of the study, which does not allow any conclusions as to the development of this deficit over time. It should be noted that when interpreting behavioral test results, Fisher 344 wildtype rats are known to deteriorate cognitively in behavioral tests as they age, and we can only measure the effect of the transgene beyond the naturally occurring cognitive decline of Fisher 344 rats [22–24]. Moreover, due to the longitudinal design of the present study, rats are repeatedly exposed to the Morris water maze test and may become familiar with it to the point where learning deficits may have been masked, thereby underestimating the cognitive deficit in the current design. Future investigations will have to clarify whether APP21 transgenic rats show impairment specific to the Water maze test, or whether other spatial tests, e.g., the Barnes maze, as well as non-spatial learning and memory tasks, such as the novel object recognition test, also precipitate a cognitive deficit in these rats. Additionally, robust tasks such as set-shifting tasks will allow for a better assessment of executive dysfunction in the APP21 rat.

In line with the naturally occurring aging process in Fisher 344 rats, both WT and TG rats showed a clear age-related progression of white matter inflammation as measured by the presence of OX-6 positive, activated microglia in the corpus callosum and in the internal capsule. Based on the assessments at 15 and 19 months of age, APP21 TG rats seem to develop this pathology earlier than WT rats, but plateau so that both genotypes demonstrate similar OX-6-positive microglia load at 19 months of age. The presence of significantly increased white matter inflammation in TG rats compared to WT rats at 15 months of age suggests that TG rats are developing histological pathology in line with a pre-dementia state in humans earlier or more readily than wildtype rats do. Combined with cognitive deficits, APP21 TG rats seem to present a unique model of pre-AD pathology. This offers a novel opportunity to study preventative strategies for dementia in a pre-clinical rat model capable of undergoing sophisticated behavioral tests [10].

A limitation to this study is that only male rats were investigated. It is possible that sexual dimorphisms exist between males and females with respect to behavioral responses as this has been reported in the past [25]. Future work could investigate any sexual dimorphisms in behavior and pathology in APP21 TG rats. Even so, the fact that APP21 TG rats are sensitive to developing more severe pathology when challenged, but only over the course of several months, also mirrors the clinical situation with regards to known non-genetic risk factors for dementia in middle age, such as smoking, stroke, and multiple concussions or traumatic brain injury [26], which have to our knowledge not been tested in this model. All these features make the APP21 TG rat a unique model for studying risk factors for AD.

## Conclusions

We here provide behavioral and histopathological evidence in support of APP21 TG rats being a novel clinically relevant model to study early correlates of cognitive impairment. Widespread, diffuse white matter inflammation may point to white matter pathology as a prominent pathological mechanism in early processes of cognitive decline. Further studies are needed to elucidate how white matter inflammation can progress to AD-like histopathology when APP21 transgenic rats are challenged, and whether counteracting this early inflammatory stage can prevent or delay neuropathology consistent with neurodegenerative disease.

## Additional file

Additional file 1: Figure S1 Morris water maze swim speed. Average speed across learning trials is not statistically different between WT and TG rats at any time point. Figure S2 Open field activity. TG animals spent significantly less time ambulating in the arena during the 10 min test period than WT counterparts at 3 months of age (A). Likewise, WT animals exhibit significantly more rearing activity (B). At 19 months, TG animals still spent significantly less ambulating than WT rats (C). Graphs show mean ± SEM. One asterisk indicates *p* < 0.05, two asterisks indicate *p* < 0.01 (Mann–Whitney test), *n* values indicated within graph bars. Figure S3 Luxol fast blue histology. Photomicrographs of coronal rat brain sections stained with Luxol fast blue from 3 months (A), 15 months (B), and 19 months (C) WT and TG rats. Higher magnification insets are from the (i) corpus callosum and (ii) internal capsule. Quantification in the anterior corpus callosum (D), posterior corpus callosum (E), and internal capsule (F) showed no significant differences in myelin content between genotypes or age time points. Graphs show mean ± SEM, *n* values indicated within graph bars. (DOCX 8940 kb)

## Abbreviations

AD: Alzheimer’s disease
APP: Amyloid precursor protein
CSS: Chaining search strategy
DIM: Direct immediate (swim pattern)
MCI: Mild cognitive impairment
TG: Transgenic
WT: Wildtype
