# Supplementary material for: APP21 transgenic rats develop age-dependent cognitive impairment and microglia accumulation within white matter tracts
Source: J Neuroinflammation. 2018 Aug 28;15:241. doi: 10.1186/s12974-018-1273-7 (PMC6114740; doi:10.1186/s12974-018-1273-7)
Supplement: Supplementary file 1 — Figure S1 Morris water maze swim speed. Average speed across learning trials is not statistically different between WT and TG rats at any time point. Figure S2 Open field activity. TG animals spent significantly less time ambulating in the arena during the 10 min test period than WT counterparts at 3 months of age (A). Likewise, WT animals exhibit significantly more rearing activity (B). At 19 months, TG animals still spent significantly less ambulating than WT rats (C). Graphs show mean ± SEM. One asterisk indicates p < 0.05, two asterisks indicate p < 0.01 (Mann–Whitney test), n values indicated within graph bars. Figure S3 Luxol fast blue histology. Photomicrographs of coronal rat brain sections stained with Luxol fast blue from 3 months (A), 15 months (B), and 19 months (C) WT and TG rats. Higher magnification insets are from the (i) corpus callosum and (ii) internal capsule. Quantification in the anterior corpus callosum (D), posterior corpus callosum (E), and internal capsule (F) showed no significant differences in myelin content between genotypes or age time points. Graphs show mean ± SEM, n values indicated within graph bars. (DOCX 8940 kb) [file 12974_2018_1273_MOESM1_ESM.docx]

**Supplemental Information**

**APP21 transgenic rats develop age-dependent cognitive impairment and microglia accumulation within white matter tracts**

Nina Weishaupt^1*^, Qingfan Liu^1*^, Sheojung Shin^1^, Ramandeep Singh^1^, Yuksel Agca^2^, Cansu Agca^2^, Vladimir Hachinski^3^ and Shawn Narain Whitehead^1^

**
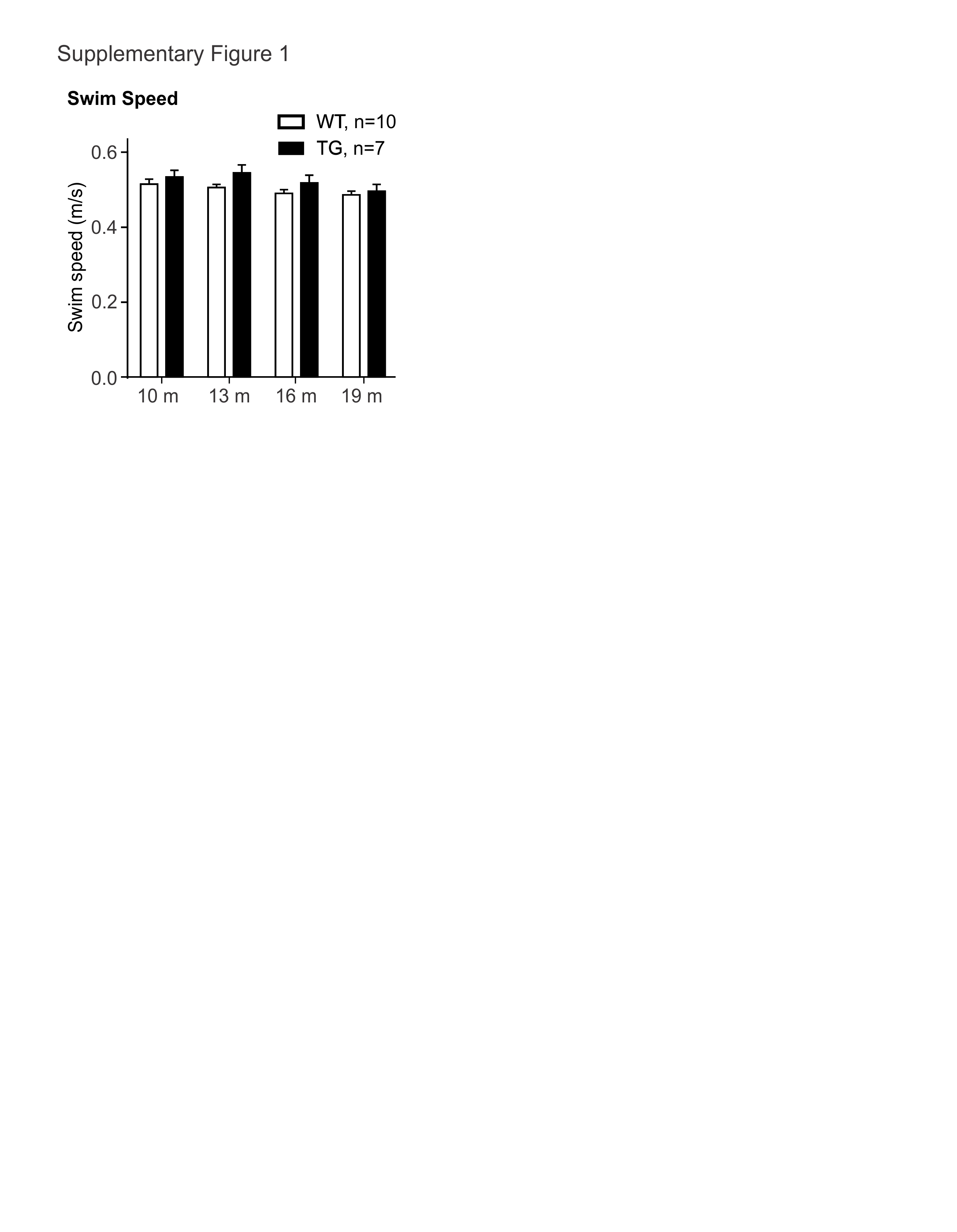
**

**Figure S1:** **Morris Water Maze swim speed.** Average speed across learning trials is not statistically different between WT and TG rats at any time point.


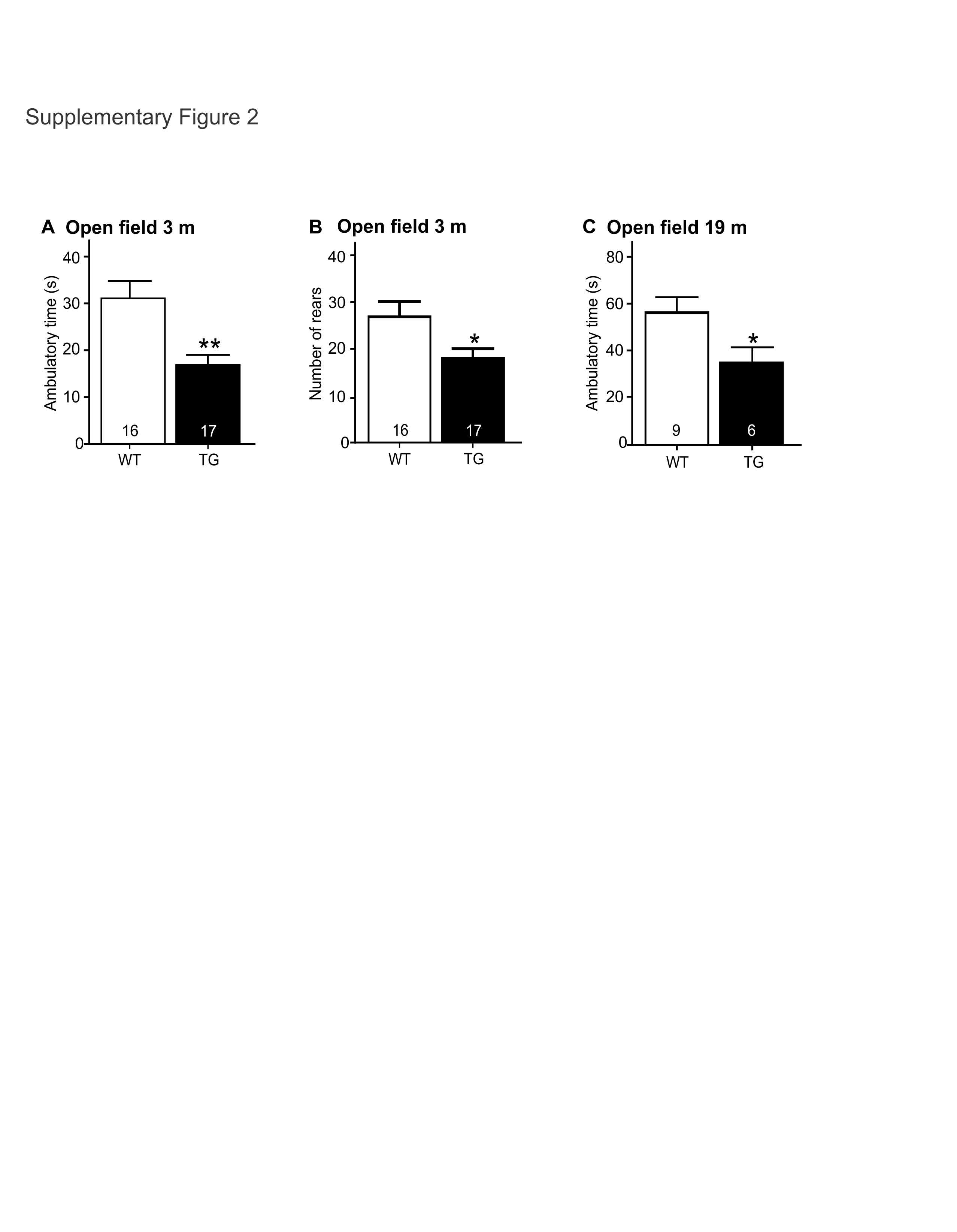


**Figure S2: Open field activity.** TG animals spent significantly less time ambulating in the arena during the 10 min test period than WT counterparts at 3 months of age (A). Likewise, WT animals exhibit significantly more rearing activity (B). At 19 months, TG animals still spent significantly less ambulating than WT rats (C). Graphs show mean ± SEM. One asterisk indicates *p*<0.05, two asterisks indicate *p*<0.01 (Mann Whitney test), n values indicated within graph bars.


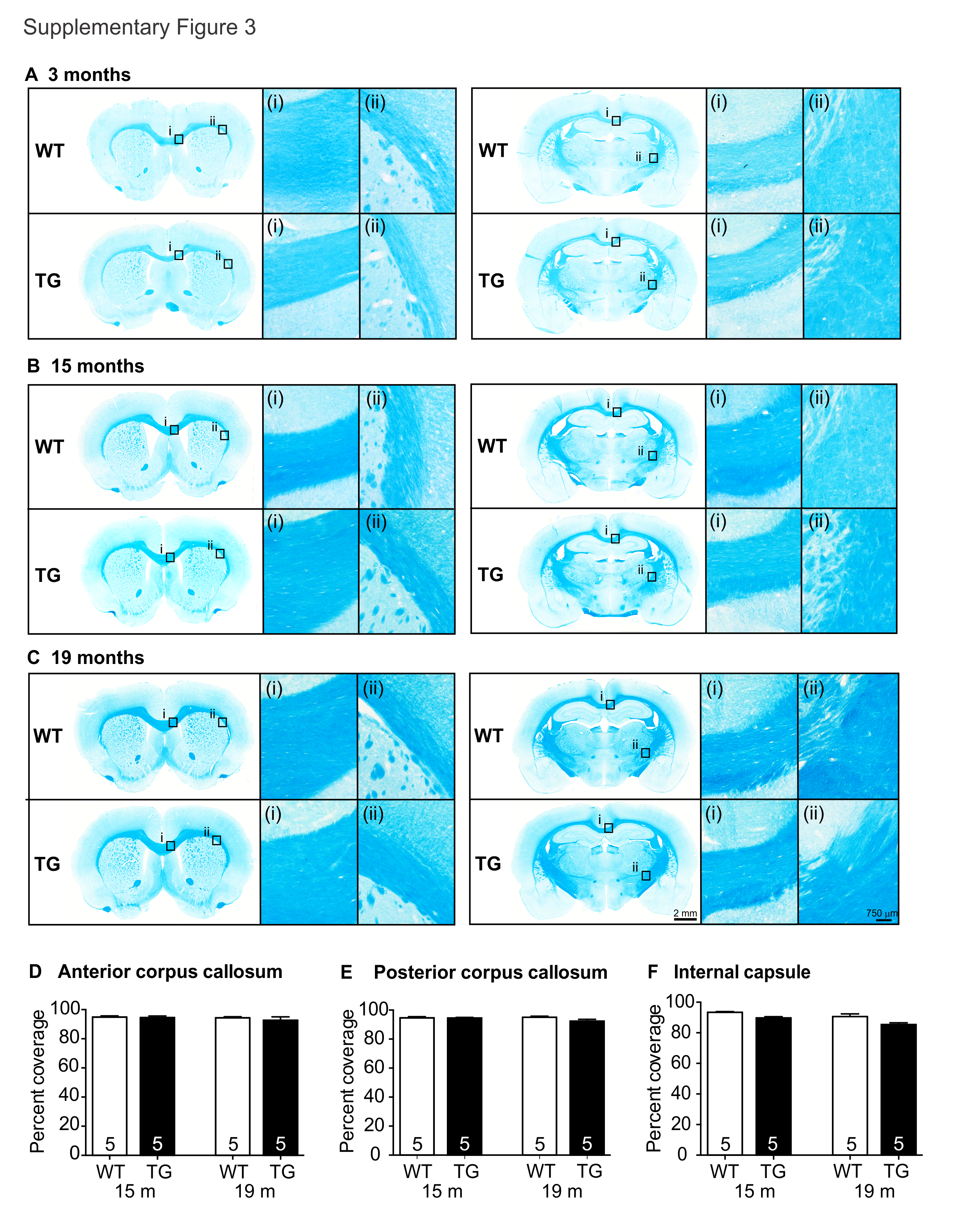


**Figure S3: Luxol fast blue histology.** Photomicrographs of coronal rat brain sections stained with Luxol fast blue from 3 m (A), 15 m (B) and 19 m (C) WT and TG rats. Higher magnification insets are from the (i) corpus callosum and (ii) internal capsule. Quantification in the anterior corpus callosum (D), posterior corpus callosum (E), and internal capsule (F) showed no significant differences in myelin content between genotypes or age time points. Graphs show mean ± SEM, n values indicated within graph bars.
